# Reassessment of contact restrictions and testing campaigns against COVID-19 via spatio-temporal modeling

**DOI:** 10.1007/s11071-021-07111-w

**Published:** 2021-12-20

**Authors:** Naleen Chaminda Ganegoda, Karunia Putra Wijaya, Joseph Páez Chávez, Dipo Aldila, K. K. W. Hasitha Erandi, Miracle Amadi

**Affiliations:** 1 Department of Mathematics, University of Sri Jayewardenepura, Nugegoda, 10250 Sri Lanka; 2 Mathematical Institute, University of Koblenz, D–56070 Koblenz, Germany; 3 Center for Applied Dynamical Systems and Computational Methods (CADSCOM), Faculty of Natural Sciences and Mathematics, Escuela Superior Politécnica del Litoral, P.O. Box 09-01-5863, Guayaquil, Ecuador; 4 Center for Dynamics, Department of Mathematics, TU Dresden, D–01062 Dresden, Germany; 5 Department of Mathematics, University of Indonesia, Depok, 16424 Indonesia; 6 Department of Mathematics, University of Colombo, Colombo, 00700 Sri Lanka; 7 Department of Mathematics and Physics, Lappeenranta University of Technology, FI–53851 Lappeenranta, Finland

**Keywords:** COVID-19, Spatial auto-correlation, Metapopulation model, Bifurcation theory, Path-following-based continuation

## Abstract

Since the earliest outbreak of COVID-19, the disease continues to obstruct life normalcy in many parts of the world. The present work proposes a mathematical framework to improve non-pharmaceutical interventions during the *new normal* before vaccination settles herd immunity. The considered approach is built from the viewpoint of decision makers in developing countries where resources to tackle the disease from both a medical and an economic perspective are scarce. Spatial auto-correlation analysis via global Moran’s index and Moran’s scatter is presented to help modulate decisions on hierarchical-based priority for healthcare capacity and interventions (including possible vaccination), finding a route for the corresponding deployment as well as landmarks for appropriate border controls. These clustering tools are applied to sample data from Sri Lanka to classify the 26 Regional Director of Health Services (RDHS) divisions into four clusters by introducing convenient classification criteria. A metapopulation model is then used to evaluate the intra- and inter-cluster contact restrictions as well as testing campaigns under the absence of confounding factors. Furthermore, we investigate the role of the basic reproduction number to determine the long-term trend of the regressing solution around disease-free and endemic equilibria. This includes an analytical bifurcation study around the basic reproduction number using Brouwer Degree Theory and asymptotic expansions as well as related numerical investigations based on path-following techniques. We also introduce the notion of *average policy effect* to assess the effectivity of contact restrictions and testing campaigns based on the proposed model’s transient behavior within a fixed time window of interest.

## Introduction

COVID-19 outbreaks have been curtailing socio-economic activities around the globe. Over 150 million total confirmed cases had been reported by Apr 29, 2021, and the number of deaths exceeded 3 million by Apr 16, 2021, reflecting the burden of the pandemic [1]. This unprecedented health crisis has shown how far time and spatial propagation of incidence matter to each individual on a micro-scale and subsequently to a country on a macro-scale. Toward the ultimate herd immunity, several vaccines have been introduced; however, their efficacy must be scrutinized amidst virus mutations [2, 3]. World Health Organization sets a minimum efficacy of 50% with a preferable threshold of 70% [4]. Although many of the vaccines are well above these efficacy levels, effectiveness in the field might be different due to the variations in affordability, public compliance, healthcare planning, etc. [5, 6]. Moreover, equitable access to vaccines, in particular for developing countries, is also a challenging task [7]. Therefore, all the non-pharmaceutical interventions (NPIs) by means of contact restrictions (physical distancing, wearing face masks, washing hands, crowd clearance, workplace clearance, school closure, lockdown, public curfew, mobility restriction), and testing campaigns (including contact tracing) must be maintained until vaccination programs take substantial control over the further spread [8–10]. Many developing countries are still subject to financial restrictions against the import of vaccines [7], and at the same time, NPIs give a variable impact due to wavering laws and public compliance that mostly weigh upon socio-economic reasons [11]. As far as the spatial aspect is concerned, these NPIs should be implemented considering disease and societal impact according to international, national, and regional epidemiological situations [12]. Research on the actual performance of NPIs in developing countries is limited, and thus related government decisions usually are over- or underestimated [13, 14]. It further creates a dilemma on what is more important between intra-regional and inter-regional contact restrictions, in particular for reopening the economy [15].

As vaccines with yet unknown success rates toward herd immunity are not even equally affordable across different economic classes, the only alternatives are enforcing laws and reshaping public awareness toward upholding NPIs. In relation to the spatial aspect, we start our investigation with the following questions:

1. On what sense may the decision maker appropriately perform the prioritization of healthcare capacity (e.g., hospital beds, ICU units, testing capacity, monitoring quarantine, including limited vaccines) among all spatial units in a country?
2. Under limited data of confounding factors, how can the decision maker value and reassess the flow of epidemics as well as the impeding NPIs?

This work puts up not only the prioritization of healthcare capacity and NPIs among spatial units but also the route for them in a more robust way than incidence-driven approaches. Endeavor to this has been known from the field of spatial mapping, namely to group spatial units into meaningful clusters.

In Sec. 2, we adopt global Moran’s index and Moran’s scatter to measure the timely spatial pattern of COVID-19 incidence in a country as well as to set the grouping. Particularly in developing countries, prioritizing high-risk areas or hotspots is driven by careful utilization of healthcare capacity [16]. The two aforementioned tools stand out among simplistic case mappings for their power to localize and group hotspots. Accordingly, priority for intra-cluster NPIs remains the same within a cluster but sequential between clusters. This strategy is important for developing countries like Sri Lanka that has not yet been covered by a holistic spatial analysis of this caliber. In addition to prioritization and route, the clustering study can bear the locations for placing border controls, which in this case are those in the main inter-cluster mobility streams. There remains, however, one caveat from these tools. That is, they are not able to parameterize the ongoing government decisions in terms of numbers and thus fail to impart how sensitive the incidence is against changes in those decisions.

Focusing on Sri Lanka, in Sec. 3 we propose a metapopulation model for Moran’s clusters determined from available panel COVID-19 incidence data. The preference of the dynamic model over functional regression models stems from integrable mechanistic processes behind COVID-19 infection and that no spatio-temporal data of confounding factors were found. A complexity reduction is proposed based on the unavailability of related field data, resulting in a simple model but rational enough such that contact restrictions and testing campaigns are mediated. Sects. 3.2–3.4 are then devoted to studying the likelihood if the incidence persists for a long time. To this, the model solution is compared with certain equilibria in the local sense whereby the basic reproduction number and effective reproduction numbers play the key role within. The model fitting as in Sec. 4 will provide a proxy to not only the approximate reproduction numbers but also non-observable dynamics, including contact matrix and the ongoing government decision on testing campaigns.

Finally, Sec. 5 extends the bifurcation analysis numerically using a path-following technique for the case where according to the fitting, the clusters are not strongly connected. In addition to this, the performance of the government decisions on contact restrictions and testing campaigns during the observations is reassessed via maximal *average policy effect*, which measures the average number of individuals per 1,000,000 inhabitants that could have been saved from COVID-19 infection on the virtue of better interventions. Scenarios to cost-to-benefit ratio are also presented alongside.

## Spatio-temporal analysis

This section is devoted to answering question 1 in the context of Sri Lanka. Particularly under consideration is prioritization of healthcare capacity and NPIs as well as classification of the 26 Regional Director of Health Services (RDHS) divisions into Moran’s clusters.

**Fig. 1:**
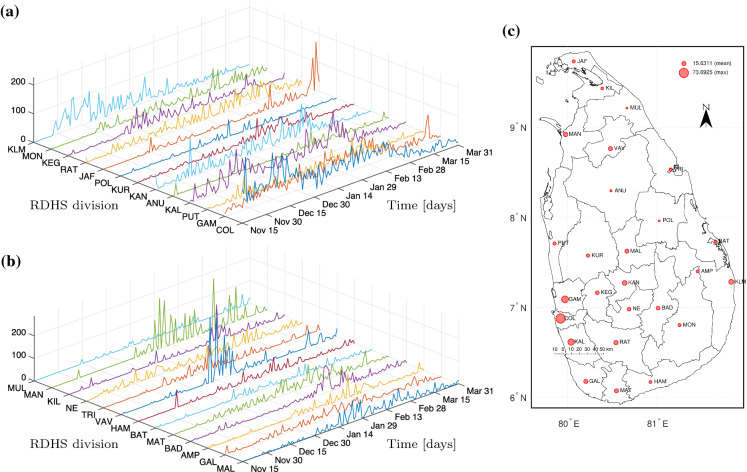
Daily COVID-19 new cases in RDHS divisions per 1,000,000 inhabitants (**a–b**) and their timely average including spatial average and maximum (**c**). The RDHS divisions are COL (Colombo), GAM (Gampaha), PUT (Puttalam), KAL (Kalutara), ANU (Anuradhapura), KAN (Kandy), KUR (Kurunegala), POL (Polonnaruwa), JAF (Jaffna), RAT (Ratnapura), KEG (Kegalle), MON (Moneragala), KLM (Kalmunai), MAL (Matale), GAL (Galle), AMP (Ampara), BAD (Badulla), MAT (Matara), BAT (Batticaloa), HAM (Hambantota), VAV (Vavuniya), TRI (Trincomalee), NE (Nuwaraeliya), KIL (Kilinochchi), MAN (Mannar), and MUL (Mullaitivu)

### Study area and observation period

Sri Lanka is a South Asian island country situated in the Indian Ocean between latitudes 5$^\circ$55’ and 9$^\circ$50’ N and between longitudes 79$^\circ$31’ and 81$^\circ$53’ E. Sri Lanka has a population of about 21.9 million [17]. From an administrative perspective, the country is divided into 9 provinces that cater to 25 districts. In health administration, there are 26 Regional Director of Health Services (RDHS) divisions that mainly coincide with administrative districts, except the district Ampara that is covered by two RDHS divisions. The primary units of health administration are called Medical Officer of Health (MOH) areas. There are 356 MOH areas wherein the health surveillance activities are carried out [18]. Over 100,000 total confirmed cases and 600 deaths had been reported in Sri Lanka by Apr 24 and by Apr 13, 2021, respectively [19]. The public has been asked to follow health guidelines such as wearing masks, washing hands, and keeping one-meter distance since the early stage of the outbreak [20]. All the confirmed cases are directed to hospitals, and close contacts in addition to overseas returnees are requested to be quarantined [20]. The data used in this study are the daily new cases recorded by the Epidemiology Unit, Ministry of Health of Sri Lanka, spanning over the period from Nov 14 until Mar 31, 2021 [21]. Recording data in RDHS level began from Nov 14, which lies within the post-curfew period after major superspreading events (apparel factory cluster [22] and fish market cluster [23]). Earlier to that, the data had been listed only according to the clusters arisen from superspreading events and quarantine centers. This is due to that only several clusters were significant rather than a community level spread up to the end of Oct 2020 [21]. The RDHS-wise normalized daily new cases (per 1,000,000 inhabitants) are illustrated in Fig. 1. Note that no major mobility restrictions had been imposed within the observation period.

### Global Moran’s index and Moran’s scatter

For spatial auto-correlation, interconnectivity between spatial units indexed by *i* and *j* is usually represented by a spatial weight matrix $W=(w_{ij})$. These weights can be designed according to shared boundaries of spatial units or distance between centers. The usual adjacency matrix can be an option, but a distance measure may better articulate connectivity since adjacency only captures interaction among neighbors. In our case, the distances $d_{ij}$ among RDHS divisions $\text {R}_i$ are based on placing appropriate centers $(x_i^{\text {R}},y_i^{\text {R}})$, which are taken from averaging those from MOH areas $\text {M}_k$, namely $(x^{\text {M}}_k,y^{\text {M}}_k)$, weighted by their population $P_k$. The centers consist of the latitude $x^{\text {M}}_k$ and longitude $y^{\text {M}}_k$ of the most attractive points, for example a city center, main administrative/commercial building, transport hub, main junction, etc. It then follows

1 $$\begin{aligned} (x_i^{\text {R}},y_i^{\text {R}}):=\frac{\sum _{k:\text {M}_k\in \text {R}_i}\left( P_k x^{\text {M}}_k,P_k y^{\text {M}}_k\right) }{\sum _{k:\text {M}_k\in \text {R}_i}P_k}. \end{aligned}$$

Now that the distances $d_{ij}$ are computable by the standard Haversine formula, we take the power functional form [24] of the weight

2 $$\begin{aligned} w_{ij}:= {\left\{ \begin{array}{ll} \dfrac{d_{ij}^{-\delta }}{\sum _{j}d_{ij}^{-\delta }}, &{} d_{ij} < d,\, i \ne j\\ 0, &{} \text {otherwise} \end{array}\right. }. \end{aligned}$$

The exponential decay parameter $\delta >0$ serves to scale the influence of the distance while the threshold distance $d>0$ cuts the inessential interconnectivity. It is important to note that sufficiently large *d* values help make *W* irreducible, i.e., all regions become *strongly connected*.

Suppose that time is frozen and the mean normalized cases over the period shown in Fig. 1 for $S=26$ RDHS divisions are reported as $C=(c_1,\cdots ,c_{S})$ with mean $\bar{c}$. Taking $Z=(z_1,\cdots ,z_S):=C-\bar{c}\mathbbm {1}$, the global Moran’s index $\mathscr {I}$ [25] with a row stochastic matrix *W* as in (2) is given by

3 $$\begin{aligned} \begin{aligned} \mathscr {I}&:=\frac{Z^\top WZ}{Z\cdot Z}=\left( \frac{Z}{\Vert Z\Vert _2}\right) ^\top W\frac{Z}{\Vert Z\Vert _2}\\&=\left( \frac{Z}{\Vert Z\Vert _2}\right) ^\top \left( \frac{W+W^\top }{2}\right) \frac{Z}{\Vert Z\Vert _2}. \end{aligned} \end{aligned}$$

The global Moran’s index basically is the Rayleigh quotient of $(W+W^\top )/2$ evaluated at *Z*, which brings the spatial autocovariance standardized by the variance of the data. The interpretation of the index usually comes in connection with the so-called Moran’s scatter $(Z/\sigma _C,WZ/\sigma _C)$ where $\sigma _C:=\sqrt{Z\cdot Z/S}$. It is quite apparent that the latter compares every spatial unit’s self-incidence magnitude against the mean with the weighted magnitudes from its corresponding neighbors as *spatial lags* of the unit. The four Moran’s clusters are then the cluster Q1 (first quadrant in 2-dimensional Euclidean space) referring to a set of spatial units of high incidence surrounded by their spatial lags of high incidence (*high–high*, *hotspots*), the cluster Q2 (second quadrant) for spatial units of low incidence surrounded by their spatial lags of high incidence (*low–high*), the cluster Q3 (third quadrant) for those of low incidence surrounded by their spatial lags of low incidence (*low–low*, *coldspots*), and the cluster Q4 (fourth quadrant) for those of high incidence surrounded by their spatial lags of low incidence (*high–low*). We obtain two facts accordingly. First, the regressing line of $(Z/\sigma _C,WZ/\sigma _C)$ that passes through the origin has the slope $\mathscr {I}$. Second, if $\lambda _{\min }$ and $\lambda _{\max }$ denote the minimum and maximum eigenvalue of the symmetric matrix $(W+W^\top )/2$, then the standard Rayleigh–Ritz (min–max) theorem (see e.g., [26] or [27]) suggests that $\lambda _{\min }:=\min _{\Vert u\Vert _2=1}u\cdot (W+W^\top )u/2\le \mathscr {I}\le \max _{\Vert u\Vert _2=1}u\cdot (W+W^\top )u/2=:\lambda _{\max }$. This gives somewhat the tightest range due to $|\lambda _{\min }|<\lambda _{\max }=\rho ((W+W^\top )/2)\le \Vert W\Vert _2\le \sqrt{\Vert W\Vert _1\Vert W\Vert _\infty }=\sqrt{\Vert W\Vert _1}\le (1+\Vert W\Vert _1)/2$. Since the diagonal entries of *W* are 0, evaluating the Rayleigh quotient at any of vectors in the standard basis of $\mathbb {R}^S$, namely $u=(0,\cdots ,0,1,0,\cdots ,0)$, yields $\lambda _{\min }\le 0$. If $\mathscr {I}\rightarrow \lambda _{\max }$, then more points are aligned with the regressing line of that slope, making Q1 and Q3 full of points leaving out Q2 and Q4 scarce. A *locally clustered spatial pattern* is then observed. If $\mathscr {I}\rightarrow \lambda _{\min }$ and in case $\lambda _{\min }<0$, then points are more concentrated in Q2 and Q4, indicating a *locally dispersed spatial pattern*. In between, under $\mathscr {I}\rightarrow 0$, there is no relation between self-incidence magnitudes and those from their spatial lags, leading to a *random spatial pattern*. We shall comment that the case $|\mathscr {I}|\le 1$ may be observed in many cases where $\lambda _{\max }\le 1$ but generally not always true. Besides assuring the upper bound 1 to any of the aforementioned bounds of $\lambda _{\max }$, sufficient conditions for this may include: *W* is symmetric (doubly stochastic) such that $\Vert W\Vert _1=\Vert W\Vert _\infty =1$, *W* and $W^\top$ commute in which case $\rho (W+W^\top )\le \rho (W)+\rho (W^\top )$ [28], and *W* is diagonalizable since then $\rho (W+W^\top )= \rho (W)+\rho (W^\top )$.

As far as Sri Lankan data are concerned, a technical question arises: which values of $\delta$ and *d* in the weight matrix are suitable for the data? We answer this question by computing the smallest absolute elasticity indices of Moran’s index on the average new cases. Now suppose that $\delta$ is decreased to a certain percentage $\varepsilon _{\delta }$ from its current value, i.e., $\delta \mapsto \delta -\varepsilon _{\delta } \delta$, where $0<\varepsilon _{\delta }\le 1$. In this way, $(\delta -\varepsilon _{\delta } \delta )/\delta =1-\varepsilon _{\delta }$ represents the total percentage post perturbation and $\varepsilon _{\delta }$ the percentage of increment. Taking this definition of $\varepsilon _{\delta }$ is more technically sound for a comparison among parameters as they may live in disparate scales. In response, $\mathscr {I}=\mathscr {I}(\delta ,d)$ also changes from its initial data in the same fashion

$$\begin{aligned} \frac{\mathscr {I}(\delta -\varepsilon _{\delta } \delta ,d)}{\mathscr {I}(\delta ,d)}&=1- \frac{\partial _\delta \mathscr {I}(\delta ,d)}{\mathscr {I}(\delta ,d)}\varepsilon _{\delta } \delta +\mathcal {O}(\varepsilon _{\delta }^2),\\ \frac{\mathscr {I}(\delta ,d-\varepsilon _{d}d)}{\mathscr {I}(\delta ,d)}&=1- \frac{\partial _d \mathscr {I}(\delta ,d)}{\mathscr {I}(\delta ,d)}\varepsilon _{d} d+\mathcal {O}(\varepsilon _{d}^2). \end{aligned}$$

For “fair” treatment, one usually designates $\varepsilon _\delta =\varepsilon _d=\varepsilon$, which is sufficiently small. Therefore, the first-order terms from the previous expressions take the role in determining which parameter, to which $\mathscr {I}$ is more sensitive. We then say $\mathscr {I}$ is more sensitive to the increase of $\delta$ than *d* in the regime

4 $$\begin{aligned} \frac{\partial _\delta \mathscr {I}(\delta ,d)}{\mathscr {I}(\delta ,d)} \delta > \frac{\partial _d \mathscr {I}(\delta ,d)}{\mathscr {I}(\delta ,d)}d. \end{aligned}$$

**Fig. 2:**
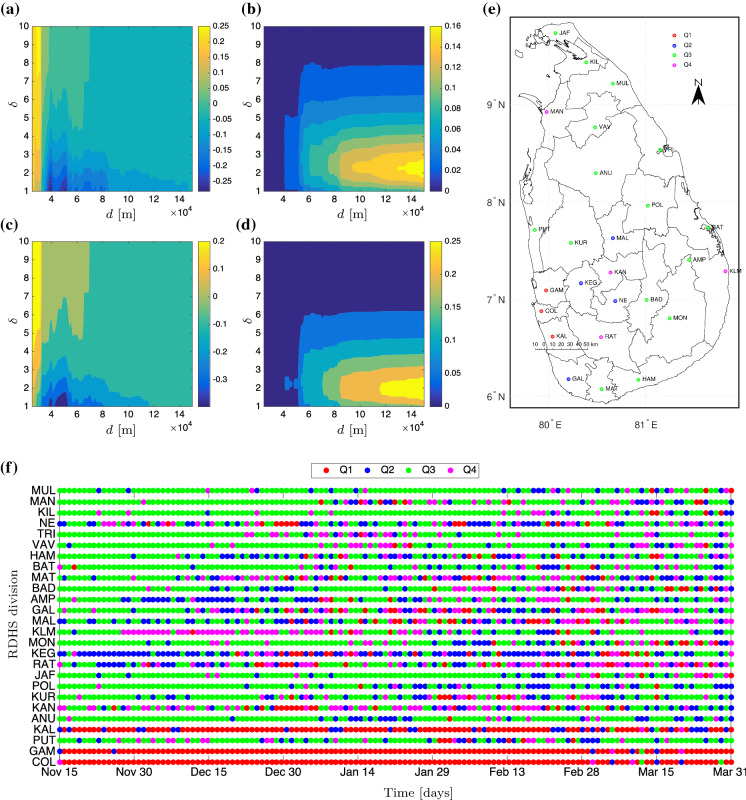
Approximates of the elasticity indices $[\mathscr {I}(d+\varepsilon d,\delta )-\mathscr {I}(d,\delta )]/[\varepsilon \mathscr {I}(d,\delta )]$ using (**a**) $\varepsilon =50\%$ and (**b**) $\varepsilon =100\%$ and $[\mathscr {I}(d,\delta +\varepsilon \delta )-\mathscr {I}(d,\delta )]/[\varepsilon \mathscr {I}(d,\delta )]$ using (**c**) $\varepsilon =50\%$ and (**d**) $\varepsilon =100\%$. Moran’s scatter plot using $d=\text {1e+05}$ m and $\delta = 9$ for the average daily new cases is presented in (**e**) while (**f**) gives the timely Moran’s scatter

In the literature, e.g., [29, 30], these two compared expressions in (4) are called the (first-order) *elasticity indices*. There is one issue, namely the non-smoothness of the index with respect to *d* limits the definition to its approximation; see Fig. 2a–d. Apparently, Moran’s index $\mathscr {I}$ is highly sensitive to *d* in case $\delta$ is relatively small ($1\le \delta \lesssim 5$) but insensitive to *d* as $\delta \gtrsim 9$. By $d=\text {1e+05}$ m and $\delta =9$, the elasticity indices are roughly zero, meaning that Moran’s index changes only very slightly under the variation of $(d, \delta )$ in a neighborhood of these values. Additionally, plotting Moran’s scatter on a daily basis (Fig. 2f) gives maximal concurrence percentages across RDHS divisions that agree with Moran’s scatter on the average data (Fig. 2e). To the latter, we obtain $\mathscr {I}\approx 0.5687$ (p value $\approx 0.000324$) indicating a locally clustered spatial pattern for the average data.

Accordingly, we classify the RDHS divisions as follows: cluster Q1 (COL, GAM, and KAL); cluster Q2 (KEG, MAL, GAL, and NE); cluster Q3 (PUT, KUR, ANU, POL, JAF, MON, AMP, BAD, MAT, BAT, HAM, VAV, TRI, KIL, and MUL); cluster Q4 (KAN, RAT, KLM, and MAN). From the application point of view, the cluster Q1 amasses all the hotspots. Ameliorating the burdens of infection follows from putting a first-level priority on healthcare capacity and possible vaccinations as well as providing more strict border controls that would reduce mobility *from and to* its spatial lags, i.e., neighbors in the sense of the weight matrix. Intra-cluster border controls cannot change the situation much, but interventions can be realized through the applications of NPIs including public curfew and testing campaigns. We argue that the intra-cluster prioritization as well as deployment route for NPIs can be left to the decision maker, which can rely on the available resources. The cluster Q4 requires not only a second-level priority on healthcare capacity but also mobility restrictions *to* its spatial lags, otherwise the epidemics outwardly *diffuses*. Meanwhile, the cluster Q2 may receive a third-level priority as well as isolation *from* its spatial lags such that it does not *attract* epidemics. Lives in the cluster Q3 can be the easiest in terms of mobility restrictions as long as reasonable hygiene practices and physical distancing are upheld. Border controls can now be localized to any point that shares the borders between clusters, which could be an intersection point on the main road or an intersecting railway station. For example, no border controls are required between KUR and ANU, but between KUR and COL. If the authorities follow a clustering based on administrative provinces, the border between KUR and ANU should be controlled as they belong to separated provinces. Thus, our analysis suggests more scientific clustering that may overrule general administrative choices.

## Modeling-based reassessment of NPIs

Now that the 26 RDHS divisions are classified into the four Moran’s clusters, this section is devoted to modeling the incidence dynamics on the clusters and parameterizing ongoing government decisions on contact restrictions and testing campaigns toward answering 1. Here is the idea: Once the essential performance measures for contact restrictions and testing campaigns are gained through the model fitting, we can optimize the model toward specific goals whereby different magnitudes of the measures are tested. We focus on three goals, namely minimizing the basic reproduction number, maximizing the average policy effect, and minimizing the associated policy cost. All forms of NPIs can be reassessed toward these goals.

The nature of standard metapopulation models suggests that, in contrast to the kinematic models, the whereabouts of every single individual are no longer concerned. Among first generations of metapopulation model, two-patch models were proposed for their accessibility to sophisticated analytical investigation on the disease endemicity via the basic reproduction number $\mathscr {R}_0$ [31–34]. These studies shared similar results: the disease-free equilibrium is globally asymptotically stable if $\mathscr {R}_0<1$, and all the state variables are uniformly persistent if $\mathscr {R}_0>1$ leading to the existence of an endemic equilibrium, which was proven to be globally asymptotically stable. The SIS model in [34] stands out among the cited models as it incorporates infections during travels. General *n*-patch SIR-type models considering mobile humans with *memory* over their origin zones admit short visits to other zones that allow them to infect other humans or to acquire infections ex-situ [35–37]. The notion of *transit time* becomes the key determinant to the latter. Models without memory were proposed in [38, 39] where in [38], a more generalized population growth function was used, taking into account the relationship between $\mathscr {R}_0$ and the disease extinction and persistence. Metapopulation models for COVID-19 have also appeared recently. Citron *et. al.* [40] consider metapopulation versions of an SIR, an SIS, and a Ross-Macdonald model integrating Eulerian movement (direct out-flux) and Lagrangian movement model (net out-flux and influx). The two movement models were analyzed to synthesize conditions under which one model can be superior against the other with respect to epidemiological outcomes. A model including transit time and infection due to exposed cases was proposed in [41] With known network attributes of the tested case, the study was brought to determine the infection rates and the ratio between asymptomatic and symptomatic cases. Recently, metapopulation models including vaccinated compartments [42] and age structure [43] were proposed and validated using field data. A memory-less migration (or diffusion) model including human mobility [44] was used for modeling daily confirmed cases on a network of 343 cities in China.

In this study, our model is concerned with the COVID-19 epidemics that naturally include undetected and deceased cases. We use the concept memory in the model, but unlike in [35–37], the number of humans from cluster *i* that are in cluster *j* and thus at which cluster the contacts happen, are not displayed. In contrast to [42], we combine the infection rate and the matrix representing the fraction of total daily time for *i*-residents to be in *j*-region into what we called a *contact matrix*.

### Metapopulation model for COVID-19 epidemics and biological assumptions

We divide the regional population $N_i$ ($i=1,\cdots ,D$) into five subpopulations based on their health status: susceptible $S_i$ (healthy but vulnerable to infection), detected *active* cases $I_i$ comprising some portions of asymptomatic and symptomatic (hospitalized) cases, undetected cases $U_i$ (*dark figures*, mostly asymptomatic), recovered $R_i$, and deceased cases $D_i$. Due to a small incubation duration, we count the intermediate exposed (pre-symptomatic) cases to the susceptible subpopulation to simplify the model presentation. Net population growth due to imports, migrations, natural births, and deaths is assumed to be negligible during the observation period, inducing constant total cluster population $N_i$. The point of departure from our modeling is concerned with

5a $$\begin{aligned} \frac{\text {d}S_{i}}{\text {d}t}&=\tilde{\mu } (S_i+I_i+U_i+R_i)-S_i\sum _{j=1}^D\tilde{\beta }_{ij} \frac{(I_j+\varrho U_j)}{N_j}\nonumber \\&\quad +\tilde{\eta } R_i-\tilde{\mu } S_i, \end{aligned}$$

5b $$\begin{aligned} \frac{\text {d}I_i}{\text {d}t}&=\alpha S_i\sum _{j=1}^D\tilde{\beta }_{ij} \frac{(I_j+\varrho U_j)}{N_j}-(\tilde{\gamma }+\tilde{\mu }) I_i, \end{aligned}$$

5c $$\begin{aligned} \frac{\text {d}U_i}{\text {d}t}&=(1-\alpha )S_i\sum _{j=1}^D\tilde{\beta }_{ij} \frac{(I_j+\varrho U_j)}{N_j}-(\tilde{\gamma }+\tilde{\mu }) U_i, \end{aligned}$$

5d $$\begin{aligned} \frac{\text {d}R_i}{\text {d}t}&=\tilde{\gamma }(1-m) I_i+\tilde{\gamma } U_i - (\tilde{\eta }+\tilde{\mu }) R_i, \end{aligned}$$

5e $$\begin{aligned} \frac{\text {d}D_i}{\text {d}t}&=\tilde{\gamma } m I_i. \end{aligned}$$

In this basic model, $\tilde{\beta }_{ij}$ denotes the infection rate that determines the likelihood of a susceptible person from *i*th region to meet with an infected person from *j*th region. In the standard SIR models for airborne diseases, the infection rates depend on many factors including sneezing rate, probability of sneezing during encounters [45], infectiousness measure (viral load, case index) [46, 47], effectiveness measure determining how probable an average susceptible person contracts infection (health condition, age) [45], human mobility for bearing corrections of the possible number of encounters [45], influence of media reports on public awareness [48], and possibly weather factors that enhance aerosol transmissions [49–51]. As the exposed cases were gathered in $S_i$, the infection rates also give another correction as the individuals cannot both be infected and spread the viruses. After contracting infection, the remaining time known as *viral shedding period* $1/\tilde{\gamma }$ determines the average duration from the onset of symptoms until the cessation of viral shedding (when viruses can no longer be released from an infected person), indicating the end of the infectiousness period [52, 53]. The parameters $1/\tilde{\eta }$ and *m* denote the duration of temporary immunity and the fatality rate from the detected cases. We impose a strong assumption that during the limited observations, the entire infected cases are timely distributed into the detected and undetected cases with the average proportions $\alpha$ and $1-\alpha$, respectively. To accommodate different transmission scales from detected and undetected cases, we have used the parameter $\varrho >1$. Finally, $\tilde{\mu }$ denotes the natural birth or death rate.

Our next task is to simplify the model even further. Due to unknown dark figures $U_i$, several ideas and estimates have been appearing in the literature, see e.g., [54]. Ours is based on the assumption that the dark figures proportionate the detected cases to a certain constant, i.e., $U_i=pI_i$ where $0<p<1$ for all clusters *i* and time. By the range of *p*, we impose that most cases are detected. As we specify $\alpha =1/(1+p)$, Eqs. (5b) and (5c) apparently become equivalent. This choice justifies the idea that the constant ratio between undetected and detected cases requires constant detection rate (in the sense of averaging) and that the detection rate also holds $0<\alpha <1$. Apart from this, we bring forward the non-observability assumption to $R_i$ due to data credibility. Our study designates $R_i$ as to proportionate $D_i$ to a certain constant from time to time, namely $R _i\simeq \eta D_i/(\tilde{\mu }+\tilde{\eta }-\eta )$ for a new parameter $0<\eta <\tilde{\mu }+\tilde{\eta }$ and all *i*. It is straightforward to see that $(\tilde{\mu }+\tilde{\eta }) R_i=\eta (R_i+D_i)=\eta [N_i-S_i-(1+p) I_i]$. In the next model presentation, we would like to use the re-scaled variables $S_i\leftarrow c S_i/N_i$ and $I_i\leftarrow c I_i/N_i$ with $c=10^6$ as well as the following notations $\mu :=\tilde{\mu }(1+p)$, $\gamma :=\tilde{\gamma }+\tilde{\mu }$, $S:=(S_1,\cdots ,S_4)^\top$, $I:=(I_1,\cdots ,I_4)^\top$, $\beta :=(\beta _{ij})$ as the *contact matrix*, $\text {diag}(S)$ as the diagonal matrix whose main diagonal is *S*, and $\mathbbm {1}$ as a matrix or a vector whose entries are 1. We acquire the final model

6a $$\begin{aligned} \frac{\text {d}S}{\text {d}t}&=\mu I-(1+\varrho p)\text {diag}(S)\frac{\beta }{c} I+\eta [c\mathbbm {1}-S-(1+p)I], \end{aligned}$$

6b $$\begin{aligned} \frac{\text {d}I}{\text {d}t}&=\frac{(1+\varrho p)}{1+p}\text {diag}(S)\frac{\beta }{c} I-\gamma I, \end{aligned}$$

with an initial value $(S_0,I_0)$. This model portrays the situation where all infected cases are distributed to the detected classes in case $p=0$, i.e., when the quality of the testing campaigns is extremely good. Moreover, as much as half of the infected cases will be distributed to the detected cases when no essential tests are done, i.e., when $p=1$. In Sri Lanka, PCR and antigen tests are carried out on a random and targeted basis [55]. However, limited financial allocations may curtail arbitrary increase in testing capacity [56]. Another factor for a large *p* is the compromised public compliance to tracing technology that tolerates the effectiveness [57]. As a result, lack of tests retards the process of unraveling possibly infected close contacts and thus hotspot identification, which eventually delays blocking the routes of transmission [58].

### Basic reproduction number

We study the basic reproduction number to determine the local behavior of model solution around the disease-free (DFE) and endemic equilibrium (EE) for fleeting observations. Therefore, given that optimal parameters are subject to data availability, the predictive power of this behavioral analysis is limited to a short-range prediction window. Let $x^*$ be a point of interest that is compared to the solution of the model $\text {d}x/\text {d}t=f(x)$ represented by (6). For simplicity, we assume that $x^*$ is either DFE or EE such that $f(x^*)=0$. The error measure $z:=x-x^*$ then follows $\text {d}z/\text {d}t= \nabla f(x^*)z+\mathcal {O}(\Vert z\Vert ^2)\approx \nabla f(x^*)z$ providing that *z* is close enough to 0 or *x* is close to $x^*$. A compelling property of such a linearized system is that the short-term trend of the model solution around 0 can be predicted by the local (even global) stability. The basic reproduction number $\mathscr {R}_0$ will then be used to parameterize a condition for the maximal real part of the eigenvalues of Jacobian matrix $\nabla f(x^*)$, which eventually determines the local stability of *z* around 0.

When $x^*=\text {DFE}=(c\mathbbm {1},0)$, we obtain

7 $$\begin{aligned} \nabla f(x^*) = \begin{pmatrix} -\eta \text {id} &{} [\mu -\eta (1+p)] \text {id} - (1+\varrho p)\beta \\ 0 &{} \frac{(1+\varrho p)}{1+p} \beta - \gamma \text {id}\end{pmatrix},\nonumber \\ \end{aligned}$$

where $\text {id}$ denotes the identity matrix. The next generation matrix as well as the basic reproduction number can now be formulated as

8 $$\begin{aligned} \begin{aligned} G&:=FV^{-1}=\frac{F}{\gamma }\\&\text {with }F:=\frac{(1+\varrho p)}{1+p} \beta ,\,V:=\gamma \text {id}\text { and}\\ \mathscr {R}_0&:=\rho (G), \end{aligned} \end{aligned}$$

respectively. Here, $\rho (G)$ denotes the spectral radius of *G*. According to Berman and Plemmons [59], $V-F$ becomes a nonsingular M–matrix if and only if $\gamma >\rho (F)$ or $1>\mathscr {R}_0$. The fact that $\lambda$ being an eigenvalue of *G* is equivalent to $\lambda -1$ being the corresponding eigenvalue of $G-\text {id}$ (with the non-changing eigenvectors), Perron–Frobenius Theorem on simplicity and dominance of $\mathscr {R}_0$ also guarantees that $\mathscr {R}_0<1$ holds true if and only if all other eigenvalues of $G-\text {id}$ (or $F-V$) lie in the open disk of center $-1$ and radius $\mathscr {R}_0$ (or center $-\gamma$ and radius $\gamma \mathscr {R}_0$). Additionally, we acquire another fact that $G-\text {id}$ (or $F-V$) becomes singular if and only if $\mathscr {R}_0=1$, in which case a zero eigenvalue occurs. Due to the equivalence relations, the final case $\mathscr {R}_0>1$ happens if and only if there exists at least one eigenvalue of $F-V$ with a positive real part. We obtain the following summary: *z* is attracted to 0 or DFE becomes locally attractive to the solution *x* of (6) if $\mathscr {R}_0<1$ and it becomes locally repelling to *x* if $\mathscr {R}_0>1$.

### Existence and attractiveness of an endemic equilibrium

Computing an endemic equilibrium (EE) from model (6) also returns complexity on its own. The second subsystem (6b) gives the equilibrium equation

9 $$\begin{aligned} S_i = \frac{(1+p)\gamma c}{(1+\varrho p)}\cdot \frac{I_i}{\sum _{j}\beta _{ij} I_j}, \end{aligned}$$

for all *j*. Substituting this to the first subsystem (6a) together with $(1+\varrho p)\text {diag}(S)\beta I/c = (1+p)\gamma I$ also multiplying every *i*-th entry with $\sum _{j}\beta _{ij}I_j/\eta c$ gives us

10 $$\begin{aligned} \begin{aligned}&\frac{[\mu -(1+p)(\gamma + \eta )]}{\eta c}I_i \cdot \sum _{j} \beta _{ij} I_j\\&\quad + \sum _{j} \beta _{ij}I_j - \frac{(1+p)\gamma }{(1+\varrho p)}I_i = 0, \end{aligned} \end{aligned}$$

for all *i*. The preceding system of equations folds under multiplication by $-(1+\varrho p)/(1+p)\gamma$ into

11 $$\begin{aligned} \begin{aligned} \varphi (I)&:= I - GI\\&\quad + \underbrace{\left[ \frac{[(1+p)(\gamma + \eta )-\mu ]}{\eta c}\cdot \frac{(1+\varrho p)}{(1+p)\gamma }\right] }_{=:K} \text {diag}(I) \beta I\\&=0, \end{aligned}\nonumber \\ \end{aligned}$$

where *G* denotes the next generation matrix as in (8). This is a multidimensional quadratic equation whose solutions cannot be derived explicitly. In order to guarantee the existence of EE, we first see if the point $(\mathscr {R}_0=1,I=0)$ is indeed a branch point of the quadratic equation. Since $\varphi :\varOmega \rightarrow \mathbb {R}^D$ is a quadratic function defined on some open subset $\varOmega \subseteq \mathbb {R}^D$, it is verifiable that $\varphi \in C^{\infty } (\varOmega )$. Let $q\in \mathbb {R}^D$ be a point such that $q \notin \varphi (\partial \varOmega )$, where $\partial \varOmega$ denotes the boundary of $\varOmega$. The point *q* is said to be *regular* if either $\varphi ^{-1}(q)=\phi$ for all points $I^*\in \varphi ^{-1}(q)$ return invertible $\nabla _I \varphi (I^*)$. Otherwise, *q* is called *critical*.

Adopting definitions from [60, 61], the map $\mathcal {B} : C^1 (\varOmega ) \times \varOmega \times \mathbb {R}^D \rightarrow \mathbb {Z}$ defined as

12 $$\begin{aligned} \mathcal {B}(\varphi , \varOmega , q) :={\left\{ \begin{array}{ll} \sum _{I^*\in \varphi ^{-1}(q)} \text {sign det} \nabla _I \varphi (I^*), &{} q \text { regular}\\ \mathcal {B}(\varphi , \varOmega , \tilde{q}), &{} q \text { critical} \end{array}\right. }\nonumber \\ \end{aligned}$$

with $\tilde{q}$ being regular such that $\Vert q-\tilde{q}\Vert <\inf _{s\in \varphi (\partial \varOmega )} \Vert q-s\Vert$, denotes the Brouwer degree of $\varphi$ in $\varOmega$ with respect to a reference point *q*. Another convention narrows the singular value down to $I=0$ with the neighborhood $\varOmega$ of 0 is chosen in such a way that $I=0$ is isolated. In this case, the map

13 $$\begin{aligned} \text {ind}(\varphi ,0) := \mathcal {B}(\varphi ,\varOmega ,0) \end{aligned}$$

defines the index of $\varphi$ at the isolated singular value $I=0$. According to the last two references, $(\mathscr {R}_0=1,I=0)$ is a branching point providing that $\text {ind}(\varphi ,0)$ changes values around $\mathscr {R}_0=1$.

In case $\mathscr {R}_0<1$, the fact that the multiplication between complex conjugate numbers return a positive number, gives us $\det \nabla _I \varphi (0) = \det (\text {id}-G) = \varPi _{i}(1-\lambda _i)>0$. We can always impose continuous perturbation on parameters $s=s(\varepsilon )\in \{\mu ,\varrho ,p,\beta _{11},\cdots ,\beta _{44},\eta ,\gamma \}$ such that *s*(0) solves $\mathscr {R}_0(0)=1$ and $s(\varepsilon )$ is equivalent to $\mathscr {R}_0(\varepsilon )>1$ for $0<\varepsilon <\tilde{\varepsilon }$ and some $\tilde{\varepsilon }$. In case $\varepsilon =0$, the eigenvalue of $\text {id}-G$ with the largest real part apparently returns $1-\mathscr {R}_0=0$ and the other eigenvalues lie in the open disk of center $-1$ and radius 1. We can appoint the eigenvalue $\hat{\lambda }$ of $\text {id}-G$ with the largest negative real part and of algebraic multiplicity $a_m(\hat{\lambda })\ge 1$, and define ${\hat{r}}:=1-\Re \hat{\lambda }$. The function $\varPhi (\lambda ,\varepsilon ):=\det ([1-\lambda ] \text {id}-G(\varepsilon ))$ with *G*(0) corresponding to $\mathscr {R}_0(0)=1$ is holomorphic in $\lambda$ and continuous in $\varepsilon$. We can appoint $r<{\hat{r}}$ such that $\hat{\lambda }$ is the only root in the closed disk $\overline{\mathbb {D}(\hat{\lambda },r)}$. There must now exist $\hat{\varepsilon }\le \tilde{\varepsilon }$ such that

$$\begin{aligned} |\varPhi (\lambda ,\varepsilon )-\varPhi (\lambda ,0)|<|\varPhi (\lambda ,0)| \end{aligned}$$

holds for all $\lambda \in \partial \mathbb {D}(\hat{\lambda },r)$ and $0< \varepsilon <\hat{\varepsilon }$. According to Rouché’s Theorem [62], $\varPhi (\lambda ,\varepsilon )$ has roots in $\overline{\mathbb {D}(\hat{\lambda },r)}$ of counting multiplicities $a_m(\hat{\lambda })$ when $0<\varepsilon <\hat{\varepsilon }$. The same continuity argument can be used to show that all the remaining eigenvalues can never have largest negative real part which exceeds $\Re \hat{\lambda }+r$. In summary, as $0<\varepsilon <\hat{\varepsilon }$ for a new $\hat{\varepsilon }$ it holds that 1 is not an eigenvalue of *G* and $\mathscr {R}_0$ slightly increases from 1 such that

14 $$\begin{aligned} \mathscr {R}_0>1>|\lambda | \end{aligned}$$

for all eigenvalues $\lambda \ne \mathscr {R}_0$ of *G*. This returns two results: (i) $\text {id}-G$ becomes non-singular such that $I=0$ serves as an isolated singular value of $\varphi$ in its neighborhood $\varOmega$ due to $\varphi (I) = \varphi (0)+ \nabla _I \varphi (0) \cdot I + \mathcal {O} (\Vert I\Vert ^2)\approx I-GI$ there and (ii) $\det \nabla _I \varphi (0) = \det (\text {id} - G) = (1-\mathscr {R}_0) \varPi _{i:\lambda _i\ne \mathscr {R}_0} (1-\lambda _i)<0$. The index of $\varphi$ at the singular value $I=0$ now reads as

$$\begin{aligned} \text {ind}(\varphi ,0) = \text {sign}\,\det \nabla _I \varphi (0) ={\left\{ \begin{array}{ll} 1, &{} \mathscr {R}_0<1\\ -1, &{} \mathscr {R}_0(\varepsilon )>1 \end{array}\right. }, \end{aligned}$$

for $0<\varepsilon <\hat{\varepsilon }$. This confirms that that $(\mathscr {R}_0=1, I=0)$ is indeed a branching point.

The next task is to verify the positivity of the local branch. We took the asymptotic expansion of $\mathscr {R}_0$ from 1 [63, 64], i.e., the coefficient of $-G$ in the quadratic equation (11) via the direct relation between $\mathscr {R}_0$ and $\varepsilon$:

15 $$\begin{aligned} 1 = \frac{1}{\mathscr {R}_0} + \frac{\mathscr {R}}{\mathscr {R}_0} \varepsilon + \mathcal {O}(\varepsilon ^2),\quad 0 < \varepsilon \ll 1 \end{aligned}$$

such that the branch *I* takes the expansion

16 $$\begin{aligned} I = \psi _1 \varepsilon + \psi _2 \varepsilon ^2 + \mathcal {O}(\varepsilon ^3). \end{aligned}$$

Substituting the preceding expressions to the quadratic equation (11) returns

$$\begin{aligned} 0&= \left[ \psi _1 \varepsilon + \psi _2 \varepsilon ^2 + \mathcal {O}(\varepsilon ^3)\right] - \left[ \frac{1}{\mathscr {R}_0} + \frac{\mathscr {R}}{\mathscr {R}_0} \varepsilon + \mathcal {O} (\varepsilon ^2)\right] \\&\,G \left[ \psi _1 \varepsilon + \psi _2 \varepsilon ^2 + \mathcal {O}(\varepsilon ^3)\right] \\&+ K\text {diag}\left[ \psi _1 \varepsilon + \psi _2 \varepsilon ^2 + \mathcal {O}(\varepsilon ^3)\right] \beta \left[ \psi _1 \varepsilon + \psi _2 \varepsilon ^2 + \mathcal {O}(\varepsilon ^3)\right] . \end{aligned}$$

Zeroing the first-order term ($\varepsilon$) gives us

17 $$\begin{aligned} G \psi _1 = \mathscr {R}_0 \psi _1. \end{aligned}$$

This means that $\psi _1$ is the eigenvector of *G* associated with $\mathscr {R}_0$, whose existence and positivity are guaranteed by Perron–Frobenius Theorem. The latter also guarantees the existence and positivity of the left eigenvector $\xi _1$ associated with $\mathscr {R}_0$. Now, multiplying the second-order term ($\varepsilon ^2$) with $\xi _1^{\top }$ from the left gives us

18 $$\begin{aligned}&\underbrace{\left[ \xi _1^{\top } - \frac{1}{\mathscr {R}_0} \xi _1^{\top } G\right] }_{=0} \psi _2 - \mathscr {R} \xi _1^\top \psi _1 \nonumber \\&\quad + K \xi _1^\top \text {diag}(\psi _1) \beta \psi _1 =0 \end{aligned}$$

whereby

19 $$\begin{aligned} \mathscr {R} = \frac{K \xi _1^\top \text {diag}(\psi _1) \beta \psi _1}{\xi _1^\top \psi _1}>0 \end{aligned}$$

by $K>0$. Moreover, substituting $(1+\varrho p)\text {diag}(S)\beta I/c = (1+p)\gamma I$ from (6b) to (6a) leads us to the following summary

20 $$\begin{aligned} \begin{aligned} S&= c\mathbbm {1}-\frac{(1+p)(\gamma +\eta )-\mu }{\eta }\psi _1\varepsilon +\mathcal {O}(\varepsilon ^2),\\ I&= \psi _1 \varepsilon + \mathcal {O}(\varepsilon ^2),\\ \mathscr {R}_0&= 1 + \mathscr {R} \varepsilon + \mathcal {O} (\varepsilon ^2). \end{aligned} \end{aligned}$$

These parametric expressions suggest that as $\varepsilon$ increases from 0, $\mathscr {R}_0$ increases from 1 and a unique local branch *I* takes off from 0 with the initial direction $\psi _1$ with respect to $\varepsilon$ whereby the susceptible state decreases from *c* simultaneously for all clusters. Finally, one yields

21 $$\begin{aligned} \begin{aligned}&\left. \partial _{\mathscr {R}_0}S\right| _{\mathscr {R}_0=1}=-\frac{[(1+p)(\gamma +\eta )-\mu ]}{\eta \mathscr {R}}\psi _1<0\\&\text {and}\quad \left. \partial _{\mathscr {R}_0}I\right| _{\mathscr {R}_0=1}=\frac{\psi _1}{\mathscr {R}}>0. \end{aligned} \end{aligned}$$

This indicates the existence of a continuum of endemic equilibria in the neighborhood of $\mathscr {R}_0=1$ and in the direction of increasing $\mathscr {R}_0$. For $0<\varepsilon \ll 1$, let us write one endemic equilibrium EE as $x^*=x^*(\varepsilon )$ with the expression given in (20). The Jacobian matrix evaluated at EE takes the form

$$\begin{aligned} \begin{aligned}&\nabla f(x^*;\varepsilon ) = \begin{pmatrix} -\eta \text {id}&{} [\mu -\eta (1+p)] \text {id} - (1+\varrho p)\beta \\ 0&{}\frac{(1+\varrho p)}{1+p} \beta - \gamma \text {id}\end{pmatrix}\\&\quad +\underbrace{ \begin{pmatrix} -(1+\varrho p)\text {diag}(\frac{\beta }{c}\psi _1)&{}(1+p)K\text {diag}(\psi _1)\beta \\ \frac{1+\varrho p}{1+p}\text {diag}(\frac{\beta }{c}\psi _1)&{}-K\text {diag}(\psi _1)\beta \end{pmatrix}\varepsilon +\mathcal {O}(\varepsilon ^2)}_{=:\mathcal {E}}. \end{aligned} \end{aligned}$$

The matrix in the leading order $\varepsilon ^0$ has eigenvalues $-\eta$ of algebraic multiplicity 4 and $\gamma (\lambda -1)$ where $\lambda$ are the eigenvalues of *G*. Due to simplicity and dominance of $\mathscr {R}_0=1$, all the eigenvalues $\gamma (\lambda -1)$ locate in the open disk of center $-\gamma$ and radius $\gamma \mathscr {R}_0$ where only $\gamma (\mathscr {R}_0-1)$ is in the origin. We can use Rouché’s Theorem one more time with the function $\varPhi (\lambda ,\varepsilon ):=\det (\nabla f(x^*;\varepsilon )-\lambda \text {id})$ to show that all eigenvalues of $f(x^*;\varepsilon )$, except the one that corresponds to $\mathscr {R}_0$, stay in the open left-half plane in $\mathbb {C}$ for a sufficiently small $\varepsilon$.

The fate of this last eigenvalue can be analyzed as follows. The eigenvalue $\gamma (\mathscr {R}_0-1)$ of $\nabla f(x^*;0)$ associates with the right and left eigenvector (by $\gamma \xi _1$ and $\gamma \psi _1$ of $\gamma G$):

$$\begin{aligned} v_L:=\gamma \begin{pmatrix} 0\\ \xi _1 \end{pmatrix}\quad \text {and}\quad v_R:=\gamma \begin{pmatrix} \frac{[\mu -\eta (1+p)]\psi _1-(1+\varrho p)\beta \psi _1}{\eta +\mathscr {R}_0}\\ \psi _1 \end{pmatrix} \end{aligned}$$

respectively. Using Taylor expansion on a simple eigenvalue of a perturbed matrix [65], we obtain the eigenvalue of the Jacobian matrix that corresponds to $\mathscr {R}_0$:

$$\begin{aligned}&\text {eig}(\nabla f(x^*;\varepsilon );\mathscr {R}_0) =\gamma (\mathscr {R}_0-1)+\frac{v_L^\top \mathcal {E}v_R}{v_L^\top v_R} +\mathcal {O}(\Vert \mathcal {E}\Vert ^2)\\&\quad \le \mathscr {R}_0-1+\frac{1}{\xi _1^\top \psi _1}(0\quad \xi _1)^{\top }\\&\qquad \begin{pmatrix} -(1+\varrho p)\text {diag}\left( \frac{\beta }{c}\psi _1\right) &{}(1+p)K\text {diag}(\psi _1)\beta \\ \frac{1+\varrho p}{1+p}\text {diag}\left( \frac{\beta }{c}\psi _1\right) &{}-K\text {diag}(\psi _1)\beta \end{pmatrix}\\&\qquad \begin{pmatrix} \frac{[\mu -\eta (1+p)]\psi _1-(1+\varrho p)\beta \psi _1}{\eta +\mathscr {R}_0}\\ \psi _1 \end{pmatrix}\varepsilon +\mathcal {O}(\varepsilon ^2)\\&\quad =\frac{K \xi _1^\top \text {diag}(\psi _1) \beta \psi _1}{ \xi _1^\top \psi _1} \varepsilon \\&\qquad -\left[ \frac{K \xi _1^\top \text {diag}(\psi _1) \beta \psi _1}{ \xi _1^\top \psi _1}+ \frac{1+\varrho p}{1+p}\xi _1^{\top }\text {diag}\left( \frac{\beta }{c}\psi _1\right) \right. \\&\qquad \left. \times \frac{[-\mu +\eta (1+p)]\psi _1+(1+\varrho p)\beta \psi _1}{\eta +\mathscr {R}_0}\right] \varepsilon +\mathcal {O}(\varepsilon ^2)\\&\quad =-\left[ \frac{1+\varrho p}{1+p}\xi _1^{\top }\text {diag}\left( \frac{\beta }{c}\psi _1\right) \right. \\&\qquad \left. \frac{[-\mu +\eta (1+p)]\psi _1+(1+\varrho p)\beta \psi _1}{\eta }\right] \varepsilon +\mathcal {O}(\varepsilon ^2) \end{aligned}$$

by substituting $\mathscr {R}_0$ from (20) and taking Taylor expansion over $1/(1+\mathscr {R}_0/\eta )$. This shows the existence of $\bar{\varepsilon }\le \hat{\varepsilon }$ where $0<\varepsilon <\bar{\varepsilon }$ implies all eigenvalues of $\nabla f(x^*;\varepsilon )$ having negative real part. Combining with (20) and (21), we acquire a forward bifurcation of the model system (6) at $\mathscr {R}_0=1$. This means that the local branch of EEs becomes locally attractive as $\mathscr {R}_0>1$.

### Effective reproduction numbers

Providing epidemics are going on, $I>0$, we have from (26):

22 $$\begin{aligned} \frac{1}{\gamma }\text {diag}(I)^{-1}I'=\frac{(1+\varrho p)}{(1+p)\gamma }\text {diag}(I)^{-1}\text {diag}(S)\frac{\beta }{c}I-\mathbbm {1}\nonumber \\ \end{aligned}$$

Observe that $I'\lesseqgtr 0$ if and only if the local *instantaneous reproduction numbers*

23 $$\begin{aligned} \mathscr {R}_i(t):=\frac{(1+\varrho p)}{(1+p)\gamma }\frac{S_i(t)}{I_i(t)}\sum _{j}\frac{\beta _{ij}}{c}I_j(t)\lesseqgtr 1 \end{aligned}$$

for all *i*. Practically speaking, if the active cases *I* determines the endemicity levels, $\mathscr {R}_i(t)$ speaks about the epidemics progression. The fact that $(1+\varrho p)\cdot \text {diag}(S)\frac{\beta }{c}I/(1+p)$ gives the inflow of new cases in all clusters, $\mathscr {R}_i(t)I_i(t)$ gives the expected number of new cases from $S_i(t)$ at the timestamp *t* attributed to the entire infected individuals from $I_j(t)$ ($j=1,\cdots ,4$) per viral shedding period $1/\gamma$. Therefore, $\mathscr {R}_i(t)$ represents the expected number of new cases from $S_i(t)$ attributed to the normalized infected individuals $I_j(t)/I_i(t)$ ($j=1,\cdots ,4$) per viral shedding period $1/\gamma$. For a realistic approximation, we took the inflow of new cases from the data while the active cases, which serve as the denominators, will be taken from the fitting. Such a definition of instantaneous reproduction number has been used by Fraser in [66], except where the active cases (as the denominator) were taken from weighted new cases in the past *n* days for a fixed *n*. The weights were later known as *serial intervals* [67, 68], estimating the distribution of delays taken from the onset of symptoms until hospital admission (i.e., when the data of new cases are usually recorded). Under the two facts that (1) the instantaneous reproduction number is, by the definition, too fluctuating and (2) infected individuals can already infect susceptible individuals from the onset of symptoms, Fraser also introduced some moving average such that the ‘real’ new cases at a certain timestamp should come from the new cases ‘recorded by hospitals’ in the future timestamps (up to *n*) weighted by the serial intervals, while the active cases come from the sum of those corresponding to the used timestamps, where again, at each timestamp the active cases are weighted sum of new cases in the past *n* days. Inspired by such refinement with, however, lack of serial interval data, ours becomes

24 $$\begin{aligned} \mathscr {R}_i(t)\approx \frac{(1+\varrho p)}{(1+p)\gamma }\cdot \frac{ \frac{1}{\tau }\int _{t}^{t+\tau }S_i(s)\sum _{j}\frac{\beta _{ij}}{c}I_j(s)\,\text {d}s}{\frac{1}{\tau }\int _{t}^{t+\tau }I_i(s)\,\text {d}s}\nonumber \\ \end{aligned}$$

for some averaging window size $\tau$. The forward moving average thus allows the serial intervals to be of uniform distribution around $\tau$ days. In the numerical computations, we designate $\tau =7$.

## Data assimilation

The basic aim of parameter estimation is to find agreement between model solution for weekly new cases $C(t_k):=\frac{(1+\varrho p)}{(1+p)}\text {diag}(S(t_k))\frac{\beta }{c} I(t_k)$ at all time points $t_k$ with known data $C^d_k$ subject to identifiability of unknown parameters $\theta =(\varrho ,p,\eta ,\beta ,S_0,I_0)$. We assume that the fitting would be subject to time-invariant i.i.d. error of the weekly covariance $\varSigma$ (for all the four clusters) and the prior was set to be Gaussian. The latter means that the parameter estimation will be based on minimizing the Mahalanobis distance between the model solution and empirical data. For simplicity, no correlation was imposed for the cluster-wise error such that $\varSigma =\text {diag}(\sigma _1^2,\cdots ,\sigma _4^2)$. The non-degenerate joint likelihood function for one time point $t_k$ is then given by

**Fig. 3:**
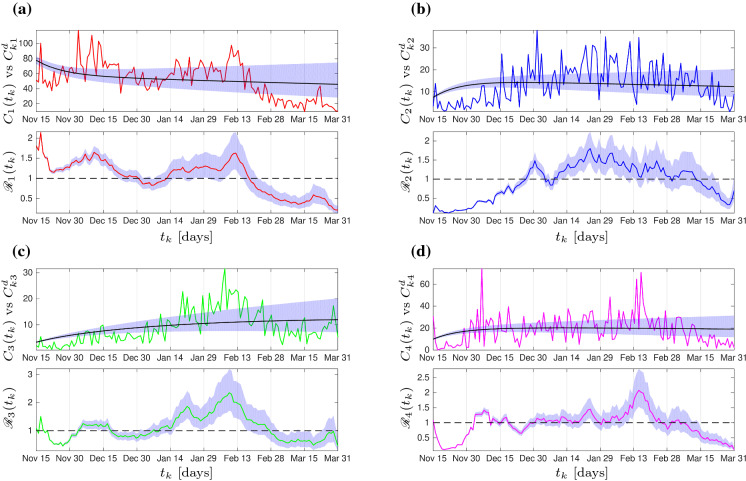
Fitting results from the deterministic SI model. The vertical axes give the numbers of weekly new cases per 1,000,000 inhabitants and effective local reproduction numbers from all clusters. The fluctuating curves represent the data and the shaded region around the fitted model solution determine the variation of $\varrho ,p,\eta$ from their confidence interval

$$\begin{aligned} \mathcal {L}_k(\theta ) := \frac{\exp \left[ -\frac{1}{2}\left( C(t_k)-C^d_k\right) ^{\top }\varSigma ^{-1}\left( C(t_k)-C^d_k\right) \right] }{\sqrt{(2\pi )^4\det \varSigma }}. \end{aligned}$$

Assuming timely i.i.d. measurement, the joint likelihood for the entire observations is then given by

$$\begin{aligned} \begin{aligned} \mathcal {L} (\theta )&:=K_G\prod _k\mathcal {L} _k(\theta )\nonumber \\&=\exp \left[ \!-\frac{1}{2}\sum _k\left( C(t_k)\!-\! C^d_k\right) ^{\top }\varSigma ^{-1}\left( C(t_k)\!-\! C^d_k\right) \right] \end{aligned} \end{aligned}$$

by taking $K_G=(2\pi )^{2|k|}(\det \varSigma )^{|k|/2}$ that serves to simplify the representation of the likelihood function [72]. Our study designates the variance terms as the mean of the data throughout the observations $(\sigma _1,\cdots ,\sigma _4)=(1/|k|)\sum _kC^d_k$ so as to avoid a blow-up in the likelihood function.

As the parameter dimension is much smaller than the data size, the standard asymptotic confidence interval [73] has been suggested to delineate the parameter uncertainty [74, 75]. The formula of the confidence interval for each optimal parameter $\hat{\theta }_\ell$ takes the form

25 $$\begin{aligned} \begin{aligned}&\left[ \hat{\theta }_\ell -\varepsilon _\ell ,\hat{\theta }_\ell +\varepsilon _\ell \right] \\&\text {where}\quad \varepsilon _\ell =\sqrt{2\chi ^2(\alpha ,df)\cdot \left( \nabla ^{-2}\log \mathcal {L}(\hat{\theta })\right) _{\ell \ell }}. \end{aligned} \end{aligned}$$

The operator $\nabla ^{-2}$ denotes the inverse of the Hessian, while the notation $\chi ^2(\alpha ,df)$ denotes the $\alpha$ quantile of the $\chi ^2$ distribution with the degree of freedom *df*. The degree of freedom can be chosen between two that further determines the type of confidence interval: $df=1$ gives *pointwise asymptotic confidence interval* (PACI) that works on the individual parameter, $df=\#\text {parameters}$ gives a *simultaneous asymptotic confidence interval* (SACI) that works jointly for all the parameters.

In the present study, the Hessian matrix in (25) will be approximated up to the second order using the queen-type stencil. Due to disparate scales of the parameters, the step size will be made dependent on the parameter’s order of magnitude, i.e., $\varDelta \theta _{\ell }:=\delta \theta _\ell$ for a uniformly small $\delta$. Our study uses $\delta \approx \text {1e--08}$. After all, the fitting will be done in MATLAB using the toolbox fmincon accompanied by interior-point as the core optimization solver. The fitting result together with the effective local reproduction numbers can be seen in Fig. 3. Meanwhile, we keep $\beta ,S_0,I_0$ at the fitted values, we vary $\varrho ,p,\eta$ from their confidence interval to have a shaded region around the fitting curves. Due to model simplicity (no time-dependent parameters), we can only expect to see an almost stationary model solution to fit the almost variance-stationary dataset, also subject to the constraint on $I_0$ of the four clusters: $I_{10}\ge I_{40}\ge I_{20}\ge I_{30}$. The fitted parameter values can be seen in Table 1.

**Table 1:** Parameters of the SI model (26). All zero $\beta$-values were due to rounding numbers smaller than 1e–07. This is intentional against floating-point error in the numerical continuation, while at the same time, almost no visible difference in the model response in comparison to that using positive values was observed

Parameter	Description	Unit	Range	Value ($\varepsilon$ PACI)	Ref.
Known:					
$1/\tilde{\mu }$	Average human lifespan	$[\text {d}]$	$70-80\cdot 365$	$76.9\cdot 365$	[69]
$1/\tilde{\gamma }$	Viral shedding period	$[\text {d}]$	$1-30$	20	[70]
$\gamma$	$\tilde{\mu }+\tilde{\gamma }$	$[\text {d}^{-1}]$			
*c*	Scaling factor	–		$10^6$	
Optimized:					
$\varrho$	Transmission scale from undetected to susceptible	–	$1-20$	11.6373 (0.6727)	
*p*	Case detection ratio	–	$0-35/65$	0.4698 (0.0148)	[71]
$\eta$	Rescaled loss-of-immunity rate	$[\text {d}^{-1}]$	$1/3\cdot 365$	4.5666e–04 (2.0482e–05)	
$\beta _{11}$	Infection rate between $S_1$ and $I_1$	$[\text {d}^{-1}]$	$0-3/c$	0.0040 (2.4694e–05)	
$\beta _{12}$	Infection rate between $S_1$ and $I_2$	$[\text {d}^{-1}]$	$0-3/c$	0.0308 (5.7954e–04)	
$\beta _{13}$	Infection rate between $S_1$ and $I_3$	$[\text {d}^{-1}]$	$0-3/c$	0 (0)	
$\beta _{14}$	Infection rate between $S_1$ and $I_4$	$[\text {d}^{-1}]$	$0-3/c$	0 (0)	
$\beta _{21}$	Infection rate between $S_2$ and $I_1$	$[\text {d}^{-1}]$	$0-3/c$	0.0032 (1.3493e–04)	
$\beta _{22}$	Infection rate between $S_2$ and $I_2$	$[\text {d}^{-1}]$	$0-3/c$	0 (0)	
$\beta _{23}$	Infection rate between $S_2$ and $I_3$	$[\text {d}^{-1}]$	$0-3/c$	0 (0)	
$\beta _{24}$	infection rate between $S_2$ and $I_4$	$[\text {d}^{-1}]$	$0-3/c$	0 (0)	
$\beta _{31}$	Infection rate between $S_3$ and $I_1$	$[\text {d}^{-1}]$	$0-3/c$	0.0012 (4.3181e–05)	
$\beta _{32}$	Infection rate between $S_3$ and $I_2$	$[\text {d}^{-1}]$	$0-3/c$	0 (0)	
$\beta _{33}$	Infection rate between $S_3$ and $I_3$	$[\text {d}^{-1}]$	$0-3/c$	0.0126 (3.0470e–04)	
$\beta _{34}$	Infection rate between $S_3$ and $I_4$	$[\text {d}^{-1}]$	$0-3/c$	0 (0)	
$\beta _{41}$	Infection rate between $S_4$ and $I_1$	$[\text {d}^{-1}]$	$0-3/c$	0.0046 (5.8647e–05)	
$\beta _{42}$	Infection rate between $S_4$ and $I_2$	$[\text {d}^{-1}]$	$0-3/c$	0 (0)	
$\beta _{43}$	Infection rate between $S_4$ and $I_3$	$[\text {d}^{-1}]$	$0-3/c$	0.0039 (4.4670e–05)	
$\beta _{44}$	Infection rate between $S_4$ and $I_4$	$[\text {d}^{-1}]$	$0-3/c$	0 (0)	
$S_{10}$	Initial condition for $S_1$	–	$0.01c-c$	8.9227e+05 (2.3154e+04)	
$S_{20}$	Initial condition for $S_2$	–	$0.01c-c$	9.3000e+05 (4.7650e+04)	
$S_{30}$	Initial condition for $S_3$	–	$0.01c-c$	6.5110e+05 (1.6834e+04)	
$S_{40}$	Initial condition for $S_4$	–	$0.01c-c$	8.1692e+05 (3.7530e+04)	
$I_{10}$	Initial condition for $I_1$	–	$0-0.1c$	569.9877 (18.4876)	
$I_{20}$	Initial condition for $I_2$	–	$0-0.1c$	569.9791 (10.1970)	
$I_{30}$	Initial condition for $I_3$	–	$0-0.1c$	37.4413 (0.8390)	
$I_{40}$	Initial condition for $I_4$	–	$0-0.1c$	569.9829 (17.9763)	
Adjusted:					
$\mu$	$\tilde{\mu }(1+p)$	$[\text {d}^{-1}]$		5.2365e–05	
$T_{\tiny \text {Ref}}$	Reference time for transient analysis	$[\text {d}]$		136	
$p_{\tiny \text {Ref}}$	Reference value for average policy effect	–		*p*	
$\omega _{\tiny \text {Ref}}$	Reference value for average policy effect	–		1	

## Numerical study of the COVID-19 model via path-following techniques

In this section, our main goal is to investigate the dynamical response of the model as certain selected parameters are varied. To evaluate the impact of reassessment on government policy against COVID-19 posterior to fitting, we shall introduce one more control parameter $\omega$ that hereafter is referred to as the *contact restriction factor*. This parameter will serve to decrease the intra- and inter-cluster contacts as so far portrayed by the fitted values of $\beta _{ij}$. From the application point of view, this parameter can be realized by enhancing NPIs and all possible interventions that likely reduce the contact between susceptible and infected persons. Furthermore, the parameter *p* (case detection ratio) will be interpreted as a factor determining the quality of COVID-19 testing campaigns in such a way that *p* close to zero represents an effective testing policy, while a large *p* indicates that a great number of infections are not detected and therefore are able to spread the disease at higher infections rates (according to the factor $\varrho$ in the SI model (26)). Consequently, the reassessment yields a small modification in the model as

26 $$\begin{aligned} \begin{aligned} \frac{\text {d}S}{\text {d}t}&=\mu I-(1+\varrho p)\omega \text {diag}(S)\frac{\beta }{c} I\\&\quad +\eta [c\mathbbm {1}-S-(1+p)I],\\ \frac{\text {d}I}{\text {d}t}&=\frac{(1+\varrho p)\omega }{1+p}\text {diag}(S)\frac{\beta }{c} I-\gamma I. \end{aligned} \end{aligned}$$

The numerical investigation will be based on the parameter fitting obtained in the previous section. There, the pair $(S_{i},I_{i})$ represents the susceptible and infected population in the cluster *i*. In this way, our study will focus on the effect of the main disease control parameters $(p,\omega )\in (0,1]^2$ on the model behavior including the basic reproduction number

27 $$\begin{aligned} \mathscr {R}_0=\frac{(1+\varrho p)\omega }{(1+p)\gamma }\rho (\beta ), \end{aligned}$$

in such a way that a fixed combination of $(p,\omega )$ will be interpreted as a specific disease control policy determined by the decision makers. The numerical study will be carried out using the path-following software COCO (Computational Continuation Core [76]). This is an analysis and development platform for the numerical treatment of continuation problems using MATLAB. A remarkable feature of COCO is its set of toolboxes that covers, to a large extent, the functionality of available continuation packages, such as AUTO [77] and MATCONT [78]. In particular, we will make extensive use of the COCO-toolbox ep, which encompasses a set of numerical routines for the bifurcation analysis of parameter-dependent families of equilibria in smooth dynamical systems.

**Fig. 4:**
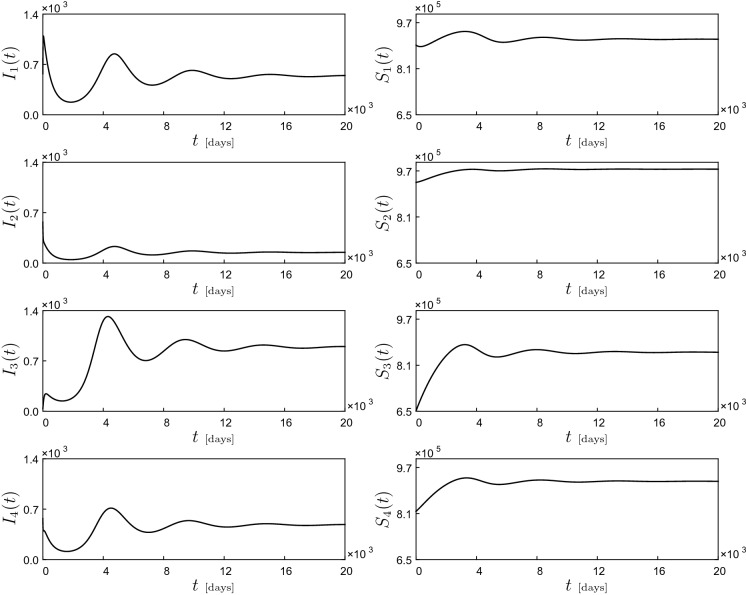
Dynamical response of the COVID-19 model (26), computed for the parameter values and initial conditions given in Table 1. The picture shows time series for the infected ($I_{i}(t)$) and susceptible population ($S_{i}(t)$)

### Monitor and cost functions

In this investigation, one of the main goals is to study the effectiveness of the disease control policy to reduce the number of COVID-19 cases in the proposed biological scenario, and for this purpose suitable performance measures will be considered in our numerical implementation. Let us assume that

$$\begin{aligned} S_{\text {\tiny Ref}}(t)\quad \text {and}\quad I_{\text {\tiny Ref}}(t),\quad 0\le t\le T_{\text {\tiny Ref}}, \end{aligned}$$

is a bounded reference solution of system (26) computed for the parameter values and initial conditions given in Table 1, with $T_{\text {\tiny Ref}}>0$ being a reference final time and $\omega =1$. In this setting, we define the performance measure given by

28 $$\begin{aligned} \begin{aligned}&M_{\text {\tiny APE}}(p,\omega ):=\\&\quad \frac{1}{T_{\text {\tiny Ref}}}\int \limits _{0}^{T_{\text {\tiny Ref}}} \left\Vert \frac{\omega _{\text {\tiny Ref}}(1+\varrho p_{\text {\tiny Ref}})}{c(1+p_{\text {\tiny Ref}})}\text {diag}\left( S_{\text {\tiny Ref}}(t)\right) \beta I_{\text {\tiny Ref}}(t)\right\Vert _{1}\,\text {d}t\\&\qquad -\frac{1}{T_{\text {\tiny Ref}}}\int \limits _{0}^{T_{\text {\tiny Ref}}} \left\Vert \frac{\omega (1+\varrho p)}{c(1+p)}\text {diag}(S(t))\beta I(t)\right\Vert _{1}\,\text {d}t, \end{aligned} \end{aligned}$$

where $\omega _{\text {\tiny Ref}}=1$ and $p_{\text {\tiny Ref}}$ is the *p*-value given in Table 1. In the above expression, *S*(*t*), *I*(*t*), $0\le t\le T_{\text {\tiny Ref}}$, stand for a solution of system (26) computed for the parameter values and initial conditions given in Table 1, but for arbitrary $(p,\omega )$. From a practical point of view, the quantity $M_{\text {\tiny APE}}(p,\omega )$ (hereafter referred to as the *average policy effect*) represents the average COVID-19 cases that could have been free from infection on a daily basis by applying a particular disease control policy $(p,\omega )$, in comparison to the reference solution case $(p_{\text {\tiny Ref}},\omega _{\text {\tiny Ref}})$ defined above. In connection to this definition, we introduce the associated *policy cost* given by

29 $$\begin{aligned} M_{\text {\tiny Cost}}(p,\omega ):=\lambda \frac{\omega _{\text {\tiny Ref}}-\omega }{\omega }+(1-\lambda )\frac{p_{\text {\tiny Ref}}-p}{p}, \end{aligned}$$

where $0\le \lambda \le 1$ is a coefficient that characterizes the cost distribution among contact restrictions and testing campaigns. As can be seen from (29), a strict mobility reduction ($\omega \approx 0$) implies a high policy cost, representing the bad impact on the economy and other negative effects associated with the mobility reduction. Similarly, a widely spread and effective COVID-19 testing campaign ($p\approx 0$) also produces very high costs, due to the personals required for implementation, expenditure on test kits and other logistics, media advertisement, organization, etc. In our investigation, the value $\lambda =0.7$ will be assigned, which portrays a realistic distribution between the two cost terms in (29) according to our numerical simulations. Nevertheless, we give such a higher contribution from contact restrictions based on bad economic impact in Sri Lanka due to job and earning losses associated with mobility restriction and crowd clearance, which additionally force the government to spend much on welfare activities targeting low-income citizens [79]. Therefore, the cost function given in (29) takes not only the view of government spending but also the economic recession in the whole country into account.

### Numerical investigation of the modified COVID-19 model

With the mathematical framework introduced in the earlier section, we can now move on to the numerical study of the modified COVID-19 model (26) using parameter values and initial conditions given in Table 1. Observe that the contact matrix $\beta$ is no longer irreducible. As a result, the initial direction of the continuum of endemic equilibria $\psi _1$ as in (20) is only guaranteed to be nonnegative according to Perron–Frobenius Theorem. A preliminary system response can be seen in Fig. 4. The picture shows time series for the active cases $I_{i}(t)$ and susceptible population $S_{i}(t)$, corresponding to Moran’s clusters Q*i* where $i=1,2,3,4$. As can be seen in the figure, for the selected parameter values the system shows a damped oscillatory behavior that settles down after a long time to an endemic equilibrium, i.e., a steady state where the COVID-19 infection is present in all clusters. This equilibrium state will then be used as starting point for our numerical investigation based on path-following techniques.

**Fig. 5:**
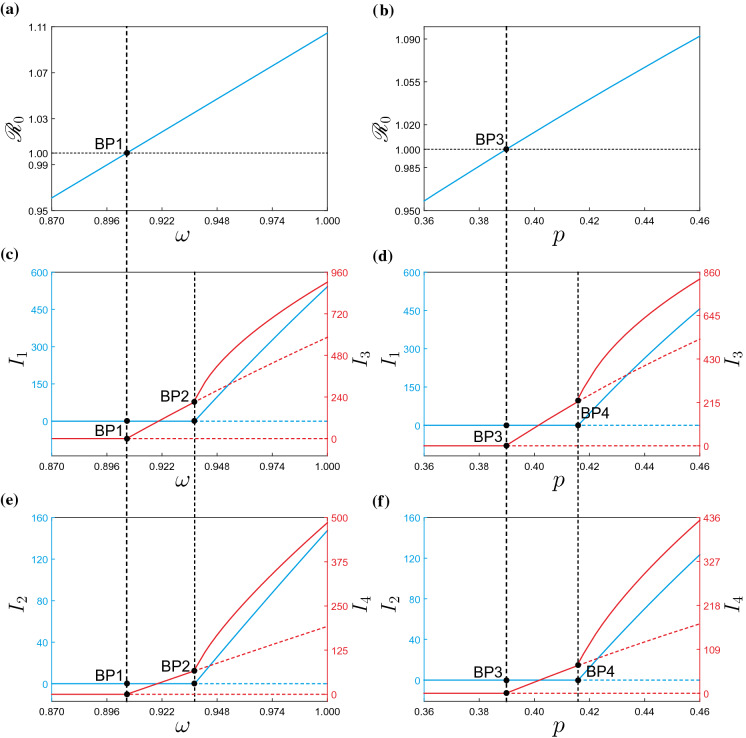
One-parameter continuation of equilibria of system (26) with respect to the mobility restriction factor $\omega$ and the case detection ratio *p*, computed for the parameter values given in Table 1. Panels **a** and **b** depict the behavior of the basic reproduction number $\mathscr {R}_{0}$ given by formula (8). Panels **c** and **d** present the behavior of $I_{1}$ (left vertical axis, in blue) and $I_{3}$ (right vertical axis, in red). Similarly, panels **e** and **f** plot the variation of $I_{2}$ (left vertical axis, in blue) and $I_{4}$ (right vertical axis, in red) with respect to the corresponding parameters. In these diagrams, solid and dashed lines stand for branches of stable and unstable equilibria, respectively. During the computations, a series of branching points are detected for $\omega \approx 0.90535$ (BP1), $\omega \approx 0.93739$ (BP2) (depicted in panels (**a**), (**c**) and (**e**)) and $p\approx 0.38920$ (BP3), $p\approx 0.41585$ (BP4) (depicted in panels (**b**), (**d**) and (**f**))

**Fig. 6:**
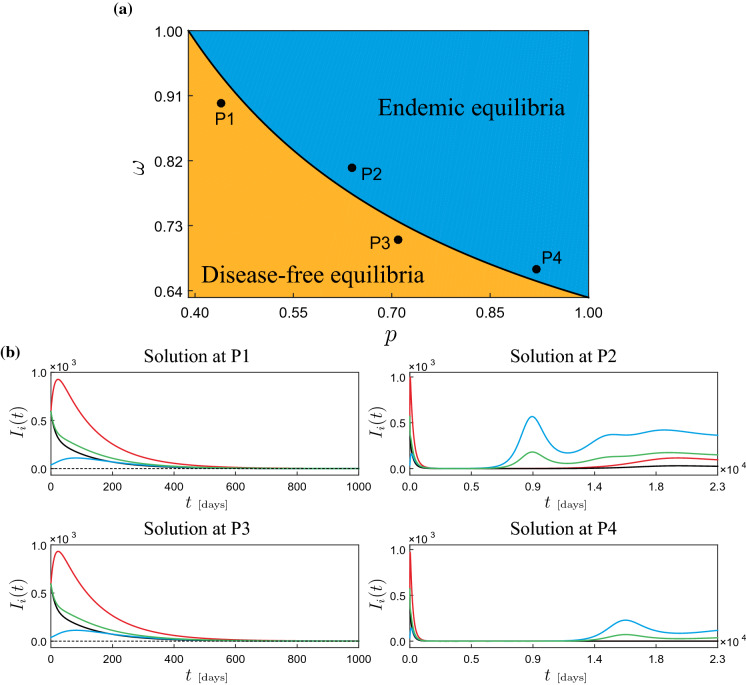
**a** Two-parameter continuation of the branching point BP1 found in Fig. 5a with respect to *p* and $\omega$. The resulting curve divides the parameter space into two regions: one for stable disease-free equilibria (yellow) and one corresponding to stable endemic equilibria (blue). **b** System responses obtained at the test points P1 ($p=0.44$, $\omega =0.90$), P2 ($p=0.64$, $\omega =0.81$), P3 ($p=0.71$, $\omega =0.71$) and P4 ($p=0.92$, $\omega =0.67$). The time plots present the behavior of $I_{1}$ (red), $I_{2}$ (black), $I_{3}$ (blue) and $I_{4}$ (green). All numerical simulations are calculated with the initial conditions specified in Table 1

**Fig. 7:**
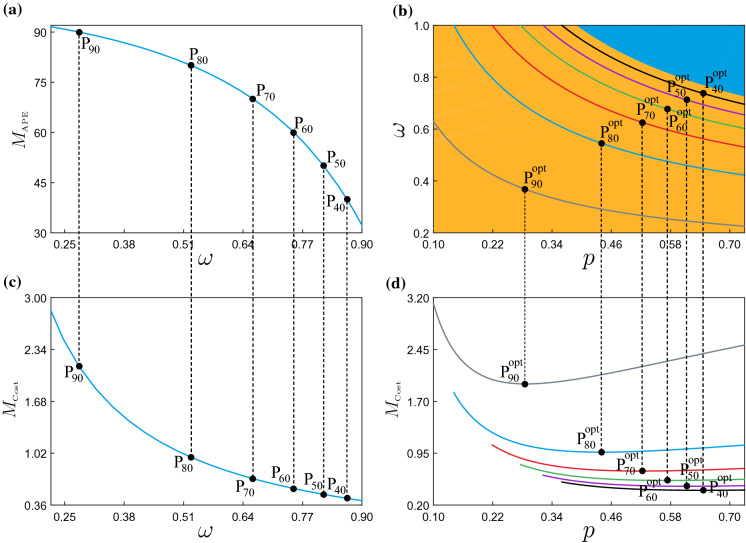
**a** One-parameter continuation of equilibria as in Fig. 5c, showing the behavior of the average policy effect $M_{\text {\tiny APE}}$ and the policy cost $M_{\text {\tiny Cost}}$ (panel **c**) with respect to $\omega$. The points P$_{L}$ correspond to $\omega$-values yielding $M_{\text {\tiny APE}}=L$. These are found at $\omega \approx 0.28137$ (P$_{90}$), $\omega \approx 0.52689$ (P$_{80}$), $\omega \approx 0.66182$ (P$_{70}$), $\omega \approx 0.75098$ (P$_{60}$), $\omega \approx 0.81627$ (P$_{50}$) and $\omega \approx 0.86723$ (P$_{40}$). **b** Two-parameter continuation of equilibria of system (26) with respect to *p* and $\omega$, keeping the average policy effect $M_{\text {\tiny APE}}$ constant (at the values specified above). In this panel, the yellow and blue regions are the same as in Fig. 6a. Panel **d** shows the behavior of the policy cost $M_{\text {\tiny Cost}}$ computed along the curves obtained in panel (**b**), for fixed $M_{\text {\tiny APE}}=90$ (grey curve), $M_{\text {\tiny APE}}=80$ (blue curve), $M_{\text {\tiny APE}}=70$ (red curve), $M_{\text {\tiny APE}}=60$ (green curve), $M_{\text {\tiny APE}}=50$ (purple curve) and $M_{\text {\tiny APE}}=40$ (black curve). In this panel, the cost for fixed $M_{\text {\tiny APE}}$ attains a minimum at $(p,\omega )\approx (0.28511,0.36847)$ (P$^{\text {\tiny opt}}_{90}$), $(p,\omega )\approx (0.43984,0.54558)$ (P$^{\text {\tiny opt}}_{80}$), $(p,\omega )\approx (0.52256,0.62619)$ (P$^{\text {\tiny opt}}_{70}$), $(p,\omega )\approx (0.57365,0.67755)$ (P$^{\text {\tiny opt}}_{60}$), $(p,\omega )\approx (0.61264,0.71263)$ (P$^{\text {\tiny opt}}_{50}$) and $(p,\omega )\approx (0.64590,0.73768)$ (P$^{\text {\tiny opt}}_{40}$)

Let us begin our study with the numerical continuation of the endemic equilibrium found above with respect to the mobility restriction factor $\omega$. The result of this process can be observed in Fig. 5, panels (c) and (e). Specifically, panels (c) and (e) present the behavior of $I_{1}$ (left vertical axis, in blue), $I_{3}$ (right vertical axis, in red) and $I_{2}$ (left vertical axis, in blue), $I_{4}$ (right vertical axis, in red), respectively, as the parameter $\omega$ varies. Panel (a) shows the dependency on $\omega$ of the basic reproduction number $\mathscr {R}_{0}$, given by formula (27). In this diagram, it can be seen that for low values of $\omega$, the basic reproduction number is smaller than one, due to which the system presents a stable disease-free equilibrium corresponding to the solid horizontal branches shown in Fig. 5c and (e). As $\omega$ increases, $\mathscr {R}_{0}$ increases as well, and it crosses 1 from below at $\omega \approx 0.90535$, where a branching point BP1 is detected. Here, the disease-free equilibrium loses stability and an endemic branch is born (via a forward bifurcation). Interestingly, at this point a COVID-19 outbreak occurs only for clusters Q3 and Q4, while clusters Q1 and Q2 remain disease-free. If $\omega$ increases further, however, the disease for clusters Q1 and Q2 develops for $\omega \approx 0.93739$, where a branching point BP2 is found. From this point onward, the disease is present in all clusters, and the increment of the infected cases augments more rapidly as the mobility restriction factor grows.

A similar scenario is encountered when the case detection ratio *p* is considered as the bifurcation parameter, see Fig. 5b, d and f. A first branching point (from below) is found for $p\approx 0.38920$ (BP3), where a COVID-19 outbreak takes place, but only for clusters Q3 and Q4, as before. A full disease development is encountered at $p\approx 0.41585$ (BP4), where now clusters Q1 and Q2 show COVID-19 infection. This scenario is clearly depicted in Fig. 5d and f showing high infections for higher *p* (i.e., for inefficient testing campaigns). Cluster-oriented interpretation can be distinguished by locally targeted testing campaigns (higher *p*) and widespread random testing campaigns (lower *p*). Our model thus conjectures that it takes smaller reduction of $(p,\omega )$ from $(p_{\text {\tiny Ref}},\omega _{\text {\tiny Ref}})$ in order to clean up the active cases in Q1 and Q2 than in Q3 and Q4.

As can be seen from the numerical study discussed above, both the mobility restriction factor $\omega$ and the case detection ratio *p* play a crucial role in controlling the disease. For instance, Fig. 5c reveals that the branching point BP1 is responsible for a first COVID-19 outbreak, occurring in clusters Q3 and Q4. Therefore, our next concern will be to investigate how this critical point varies in the *p*-$\omega$ control space. For this purpose, we will carry out a two-parameter continuation of this critical point, see Fig. 6a. The black curve represents a locus of branching points on the *p*-$\omega$ plane. The resulting curve divides the control space into two regions: one for stable disease-free equilibria (yellow) and one corresponding to stable endemic equilibria (blue). In this way, for a specific disease control policy represented by the pair $(p,\omega )$, we can determine *a priori* whether the policy will be effective or not in controlling a COVID-19 outbreak. This can be verified at the test points P1–P4 shown in Fig. 6a. For all these points, test trajectories are calculated using the data shown in Table 1, see Fig. 6b. As can be seen, the solutions computed at P1 and P3 (disease-free region, in yellow) decay to zero, while those computed for P2 and P4 (endemic region, in blue) settle down to an endemic equilibrium, where a long-term COVID-19 outbreak occurs.

### Optimization of the disease control policies

In the previous section, we applied numerical continuation methods to study the effect of the mobility restriction factor $\omega$ and the case detection ratio *p* on the behavior of the modified COVID-19 model (26). In this way, we established critical values of the control parameters upon which a disease outbreak occurs. In this section, we will consider the effect of the control parameters on the average policy effect and the policy cost, as defined in Sec. 5.1. For this purpose, we will assume that the disease control policies represented by the pair $(p,\omega )$ are chosen from the yellow region in Fig. 6a.

To begin our study, we will carry out the numerical continuation of disease-free equilibria of model (26) with respect to $\omega$ and monitor the behavior of the average policy effect $M_{\text {\tiny APE}}$ defined in (28). The result of this procedure can be seen in Fig. 7a. As can be expected, $M_{\text {\tiny APE}}$ is a decreasing function of $\omega$, since the average COVID-19 infections that can be avoided (as explained in Sect. 5.1) decrease if higher degrees of mobility between clusters are allowed. Moreover, panel (a) shows a series of points labeled P$_{L}$, which correspond to $\omega$-values yielding $M_{\text {\tiny APE}}=L$. At these points, the resulting costs are shown in Fig. 7c, which depicts the behavior of the cost function $M_{\text {\tiny Cost}}$ (see (29)) with respect to $\omega$. As can be seen in the diagram, this function grows as $\omega$ decreases, which is consistent with the fact that stricter contact restrictions lead to higher policy costs. This observation then raises the question if for a desired fixed value of $M_{\text {\tiny APE}}$, a more convenient control policy $(p,\omega )$ can be found in terms of cost reduction. To tackle this question, we will employ two-parameter continuation with respect to *p* and $\omega$ to find loci of control points $(p,\omega )$ yielding fixed values of $M_{\text {\tiny APE}}$, monitoring the corresponding cost function. The result can be seen in Fig. 7b. Here, a family of curves in the *p*-$\omega$ plane are shown for which $M_{\text {\tiny APE}}$ is kept fixed. Panel (d) presents the behavior of the cost function $M_{\text {\tiny Cost}}$ along the curves obtained in panel (b). As can be seen, in all cases the cost function presents local minima, which can be interpreted as an optimal policy implementation for a desired fixed value of $M_{\text {\tiny APE}}$.

## Concluding remarks

Analysis in this study covers spatio-temporal aspects of COVID-19 transmission in Sri Lanka with the aid of basic reproduction number and path-following continuation pertained to the role of NPIs (contact restrictions and testing campaigns). The daily new cases have been widely used data; however, we processed normalized cases via the populations of RDHS divisions. It suppresses unbiased estimates as higher numbers of cases are reported in highly populated RDHS divisions. Subsequently, all RDHS divisions were categorized using Moran’s scatter into four clusters. Prioritization as well as route for interventions should be Q1 (high-high), Q4 (high-low), Q2 (low-high), and Q3 (low-low). One useful contribution is that the government can use such a route in vaccination programs started at the latter stage of the study period. Priority within a cluster may rely on logistics available within that cluster and temporary shift from the other clusters. Our result is also helpful in placing appropriate border controls for the sake of curtailing transmission waves. Even though Q1 and Q3 do not encounter different incidence levels in their spatial lags, Q2 is vulnerable for significant absorption while Q4 is responsible for significant diffusion. Therefore, border controls can be placed in every important intersecting point between two different clusters.

We extend the qualitative clustering analysis into a quantitative one by conducting an inverse problem using the cases data. A preliminary model of SIURSD type is proposed, carrying the metapopulation context with memory. Due to non-observable model variables, dimensional reduction leads us to an SI type. This final model may look parsimonious; however, it still explains essential mechanistic processes of COVID-19 transmission: cluster-wise contact matrix, viral shedding period, transmission scaling between detected and undetected cases, case detection ratio, contact restriction factor, and loss of immunity. Fitting to the data was done to reveal hidden dynamics including contract matrix, initial conditions for the active cases, case detection ratio, transmission scaling, and loss of immunity. Nonetheless, the SI type may provide beneficiary to big data analytics, especially when the observation period and network size are extended. Forward bifurcation for strongly connected network among clusters was found around the basic reproduction number 1. Numerical investigation was done for the case where the network is, according to rounding small $\beta$-values to zero, not strongly connected. Time-varying effective local reproduction numbers for all clusters are also presented. Their appearance supersedes clueless cases data when it comes to localizing time at which the current transmission is high (reproduction number greater than 1), suggesting for immediate interventions.

**Fig. 8:**
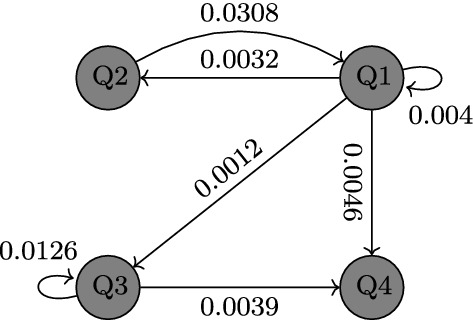
Network based on the contact matrix $\beta$. The arrow directed from the cluster Q*i* to the cluster Q*j* translates the statement “the susceptible humans in Q*j* contract infection through contact with infected humans in Q*i*” or shortly “Q*i* causes infection in Q*j*”

An interesting result is evident from one-parameter continuation of equilibria. Recalling the analytical framework in Sec. 3.3, the initial direction of the continuum of endemic equilibria at $\mathscr {R}_0=1$ is the Perron vector of the next generation matrix $\psi _1$ (see Eqs. (20) and (21)). As the network associated with the next generation matrix or the contact matrix $\beta$ is not strongly connected (see Fig. 8), Perron–Frobenius Theorem (cf. [80]) only guarantees the nonnegativity of the Perron vector. Particularly to our case, we obtain

$$\begin{aligned} \psi _1\approx \begin{pmatrix} 0\\ 0\\ 0.9553\\ 0.2957 \end{pmatrix}. \end{aligned}$$

This Perron vector indicates two findings: (1) the clusters Q1 and Q2 remain in the disease-free states when $\mathscr {R}_0$ shortly exceeds 1; meanwhile (2) the long-term number of active cases in the cluster Q3 jumps to larger extent than that in the cluster Q4 as observed in Fig. 5. If we read the bifurcation diagrams backward in $\omega$ and *p*, then these findings mean that Q1 and Q2 achieve disease-free states quicker than Q3 and Q4 under the reduction of *p* and $\omega$ from $p_{\text {\tiny Ref}}\approx 0.4698$ and $\omega _{\text {\tiny Ref}}=1$, respectively. The network in Fig. 8 explains that Q2 receives a relatively small “injection” from Q1 but returns with a large injection to Q1; meanwhile there is no essential self-injection in Q2. Equipped with a small self-injection, Q1 also injects Q3 and Q4 at comparable rates. Meanwhile, Q3 admits a relatively large self-injection but spares an injection to Q4. On the overall picture, it is arguable that Q1 and Q2 lose endemicity faster than Q3 and Q4 if the entire injection rates (the nonzero entries of the contact matrix $\beta$) are reduced simultaneously. At a certain stage, there comes, on the one hand, a scenario where the self-injection in Q1 and thus the injection to Q2 are negligible, making Q2 non-reproductive. On the other hand, the negligible injection from Q1 is compensated by the self-injection in Q3 that withstand both Q3 and Q4 in the endemic states. Notwithstanding this interesting finding, we also observe that disease-free equilibrium (DFE) can be found by reducing *p* and $\omega$ not so far away from $p_{\text {\tiny Ref}}$ and $\omega _{\text {\tiny Ref}}$, respectively. Thus, we argue that the original interventions imposed by the government had been satisfactory during the observation period. From the point of view model transients, one should note that significant contact restrictions are both costly and not gainful in terms of average policy effect (APE). This is evident by concave behavior of APE and convex behavior of the cost against $\omega$ (see Fig. 7(a,c)). Therefore, reducing *p* and $\omega$ to arbitrarily small values does not make much sense. Scenarios for the optimal values of *p* and $\omega$ minimizing the cost under fixed magnitudes of APE were proposed. As expected, even optimal results come with a price, as the optimal $(p,\omega )$-values walk toward the third quadrant by increasing APE values; see Fig. 7(b).

Finally, this study leaves us some gaps for further improvement. First, several attributes in the original model can be modified to capture more complexities. For example, the average viral shedding period $1/\tilde{\gamma }$ for the undetected cases could have been different from that of the detected cases due to nonoccurrence of symptoms. Despite the averaging, taking the timely proportion of detected cases $\alpha$ to be a constant can be too stringent owing to the unknown dark figures (undetected cases). Future improvement may include time-dependent noise for such parameters with given (under guidance of field experts) or computationally tested priors. That recovered and deceased cases preserve a constant ratio is also worth of improvement. Second, the control parameters $\omega$ and *p* actually represent adjustment of contact restrictions and testing campaigns on the national level, meaning that the scaled susceptible cases in all clusters are enforced the same way toward endemicity reduction, irrespective of their local resources. Meanwhile, the reduction of $\beta$-values via the cluster-independent $\omega$ also serves as another limitation of the model. From the application point of view, this means that all actions entailed in the contact restrictions should simultaneously follow the adjustment of $\omega$ without proper consideration as to what actions are paramount among the others. For example, reduction of $\omega$ from $\omega _{\text {\tiny {Ref}}}=1$ to 0.75 means that those who go out for activities should reduce the intensities to $75\%$, those who travel across clusters 4 days a week should change to 3 days a week, schools that are opened 4 days a week should be opened 3 days a week instead, working in the office 5 days a week should change to 3.75 equivalent working days, etc. Technically speaking, these changes may sound simple from the standpoint of the decision maker; however, different abilities and preferences among humans can make the implementation difficult to trace. Third, our SI model contains several parameters that multiply with others. A parameter identification analysis is worth considering if one were to reveal possible dependency among them and thus correct the model specification. Fourth, had regional data of confounding factors been there, we could have integrated these data e.g., in the $\beta$-values from time to time to capture the different cluster peaks and fluctuations. This is due to the fact that the $\beta$-values appear to be equivalent to the term of new cases. Incidence and meteorological data from other countries, for example, could be helpful toward this direction.

## Data Availability

Data are available upon request, which can be directed to the corresponding author.
